# Soluble HLA-I and HLA-II Molecules Are Potential Prognostic Markers of Progression of Systemic and Local Inflammation in Patients with COPD

**DOI:** 10.1155/2018/3614341

**Published:** 2018-11-27

**Authors:** Nailya Kubysheva, Svetlana Soodaeva, Viktor Novikov, Tatyana Eliseeva, Timur Li, Igor Klimanov, Elena Kuzmina, Héctor Baez-Medina, Valery Solovyev, Dmitry Yu. Ovsyannikov, Ildar Batyrshin

**Affiliations:** ^1^Kazan Federal University, Kremlyovskaya St. 18, Kazan 420000, Russia; ^2^Pulmonology Research Institute, 11-Parkovaya 32, Moscow 105077, Russia; ^3^Lobachevsky State University of Nizhny Novgorod, Gagarina Avenue 23, Nizhny Novgorod 603950, Russia; ^4^Federal State Budgetary Educational Institution of Higher Education, Privolzhsky Research Medical University, Minin and Pozharsky Square 10/1, Nizhny Novgorod 603005, Russia; ^5^Central Clinical Hospital of RAS, Litovskiy Blvd. 1A, Moscow 117593, Russia; ^6^Centro de Investigación en Computación, Instituto Politécnico Nacional (CIC-IPN), Av. Juan de Dios Bátiz, Esq. Miguel Othón de Mendizábal S/N, Gustavo A. Madero, 07738 Mexico City, Mexico; ^7^Medical Institute, Peoples' Friendship University of Russia (RUDN University), 6 Miklukho-Maklaya St., Moscow 117198, Russia

## Abstract

Soluble molecules of the major histocompatibility complex play an important role in the development of various immune-mediated diseases. However, there is not much information on the participation of these proteins in the pathogenesis of chronic obstructive pulmonary disease (COPD). The aim of our work was to determine the content of soluble molecules of the major histocompatibility complex of classes I and II (sHLA-I and sHLA-II) in the exhaled breath condensate (EBC) and in the blood serum in patients with moderate to severe COPD during the exacerbation and stable phase. We investigated 105 patients (male) with COPD aged 46–67 and 21 healthy nonsmoking volunteers (male) comparable in age. The content of sHLA-I and sHLA-II molecules was studied using ELISA. We found an increase in the level of sHLA-I and sHLA-II molecules in EBC, as well as an enhancement in the serum content of sHLA-II in all the examined COPD patients compared to healthy nonsmoking volunteers. The revealed negative correlation between the serum concentration of sHLA-II and values of FEV_1_ and FEV_1_/FVC in all examined patients with COPD gives a possibility to consider the content of these proteins as an additional systemic marker of disease severity. The maximum endobronchial and serum concentrations of sHLA-I and sHLA-II were detected in patients with severe COPD during the exacerbation. The negative associations between the content of these molecules in EBC and serum and the parameters of lung function in patients with severe COPD were established. These findings suggest a pathogenetic role of sHLA-I and sHLA-II molecules in the mechanisms of the development and progression of local and systemic inflammation in COPD.

## 1. Introduction

The basis of the pathogenesis of COPD is a progressive inflammatory process in the respiratory tract defining clinical and functional displays of this disease. Numerous cells of the immune system are involved in the inflammatory process in this disease. An activation of these cells is characterized by producing a large number of mediators, changing of the expression of membrane molecules, and secretion of soluble differentiation molecules into extracellular space [1–3].

Soluble forms of membrane proteins are endogenous regulatory molecules that are included in the global immunological network and participate in the mechanisms of the immune response at various stages of its realization [4]. Currently, the prognostic, monitoring, and diagnostic significance of various soluble forms of differentiation molecules in immune-mediated diseases of different genesis, including COPD, is being studied [5–10].

An important role in the regulation of the immune response belongs to the molecules of the major histocompatibility complex of class I (HLA-I) and class II (HLA-II). These proteins are involved in the interaction of all types of immune cells of the body, recognition of both their own, including modified ones, and foreign cells. HLA molecules can simultaneously be present both in a membrane-bound form on the cell surface and in a soluble form in various biological fluids [11, 12]. Soluble HLA proteins are formed by the proteolytic shedding of membrane proteins and/or by an alternative splicing of matrix RNA.

Significant amounts of sHLA-I and sHLA-II molecules are produced by various activated cells of the immune system under the influence of mitogens and various antigens [12–14]. Thus, the content of sHLA-I and sHLA-II can reflect the functional state of cellular immunity.

The interaction of sHLA-I and sHLA-II proteins with their physiological ligands is one of the key mechanisms regulating the action of these molecules. Soluble molecules of the major histocompatibility complex competing with their membrane-bound homologues modulate the reactivity of cellular immunity [15–17].

sHLA-I and sHLA-II have been shown to be markers of various pathological conditions and the nature of the change in their concentration may be of prognostic significance in many diseases. A change in the concentration of sHLA-I molecules was observed in many infectious, inflammatory, oncologic, and autoimmune diseases, including systemic lupus erythematosus, rheumatism, and autoimmune hepatitis [18–26]. The role of sHLA-II in the development of a number of immune-mediated human diseases such as anterior uveitis, systemic lupus erythematosus, rheumatoid arthritis, sepsis, and HIV has been demonstrated [27–32].

There is little information on the participation of sHLA-I and sHLA-II molecules in the pathogenetic mechanisms of COPD. Previously, when studying changes in the level of sHLA-I molecules in patients with an exacerbation of COPD, we observed an increase in the content of these soluble membrane proteins in severe disease degree [8]. However, there is no data on the comparative analysis of changes in the content of sHLA-I depending on the disease period (exacerbation or stable phase) in patients with different COPD severities. Currently, the role of sHLA-II in the development of COPD is also insufficiently studied. An increase in the concentration of sHLA-II in patients with COPD with antitrypsin-alpha-1 deficiency was demonstrated [33].

For a more complete understanding of the pathogenetic mechanisms of COPD progression, there is a need to determine the character of the relationship between the concentrations of sHLA-I and sHLA-II molecules and violations of lung ventilation that will allow clarifying the role of these proteins in the formation of clinical and functional disorders in this disease.

Given that inflammation in COPD has a local and systemic nature, it is important to study biomarkers simultaneously in the respiratory tract and in circulation.

The aim of this study was to evaluate the content of sHLA-I and sHLA-II molecules in the exhaled breath condensate and blood serum and determine the relationship between the concentration of these molecules and lung function parameters in patients with moderate to severe COPD during the exacerbation and stable phase of the disease.

## 2. Materials and Methods

The study included 126 people. The control group consisted of healthy nonsmoking volunteers (*n* = 21).

Patients with COPD (*n* = 105) were divided into four groups: patients with moderate COPD (GOLD II) during the exacerbation (*n* = 25), patients with moderate COPD (GOLD II) in the stable phase (*n* = 27), patients with severe COPD (GOLD III) during the exacerbation (*n* = 29), and patients with severe COPD (GOLD III) in the stable phase (*n* = 24).

Written informed consent was obtained from all participants. The study was approved by the Ethics Committee of the Pulmonology Research Institute, Moscow, Russia.

The diagnosis of COPD was defined and classified according to the criteria of the Global Initiative for Chronic Obstructive Pulmonary Disease (GOLD) [34].

The study included patients with COPD meeting the following inclusion criteria: age over 40 years, active or ex-smokers (smoking index (IC) ≥10 pack-years), acute exacerbation of COPD (AE-COPD) and stable course, evidence of obstructed lung function (postbronchodilator FEV_1_<80 %, =30 % and FEV_1_/FVC < 70%) according to the GOLD (2017) [34]. An acute exacerbation was defined as a change in the symptoms of a cough, expectoration, and dyspnea that was beyond the daily variation and required changes in therapy in patients with COPD.

The exclusion criteria were the following: asthma and other allergic diseases, pneumonia, history of congestive heart failure, severe arterial hypertension, diabetes, and conditions requiring the long-term use of systemic corticosteroids.

Pulmonary function study was carried out on a computer Spirograph “SpiroLab III” (Italy) for the evaluation of the FEV_1_, FEV_1_/FVC, and the parameters of inspiratory capacity (IC). The demographic and functional characteristics of the COPD patients are presented in Table 1.

### 2.1. Serum and Exhaled Breath Condensate Preparation

The blood samples were obtained in the morning on an empty stomach from the median cubital vein, immediately centrifuged at 3000 rpm for 10 minutes, and then extracted.

The exhaled breath condensate was collected within 20 minutes after rinsing the mouth using the RTube system (Respiratory Research, Inc., USA).

The serum and EBC were stored at *T* = −80°C.

### 2.2. Measurement of the Content of Soluble HLA-I and sHLA-II Molecules

The concentrations of sHLA-I and sHLA-II molecules in serum and EBC were determined by an enzyme-linked immunosorbent assay (ELISA) using an ELISA reader (Multiskan MS, LabSystems, Finland) at a wavelength of 405 nm.

In determining the content of sHLA-I molecules, we used monoclonal antibodies ICO-53 and ICO-216 conjugated with horseradish peroxidase. The concentration of sHLA-II molecules was examined by using monoclonal antibodies ICO-1 and goat polyclonal antibodies against HLA-II molecules conjugated with horseradish peroxidase as described in [25]. The results were expressed in conventional units (U/ml).

### 2.3. Statistical Analysis

The statistical analysis was carried out using the Statgraphics Centurion software package, v.9. The data were presented as the mean ± SD. To determine the distribution normality, the Shapiro-Wilk test was used. Further analysis was performed by ANOVA analysis and Student's *t*-test. To calculate the correlation coefficient (*r*) the Pearson correlation test was used. The statistical significance level was considered to be *p* < 0.05.

## 3. Results

**Figure 1:**
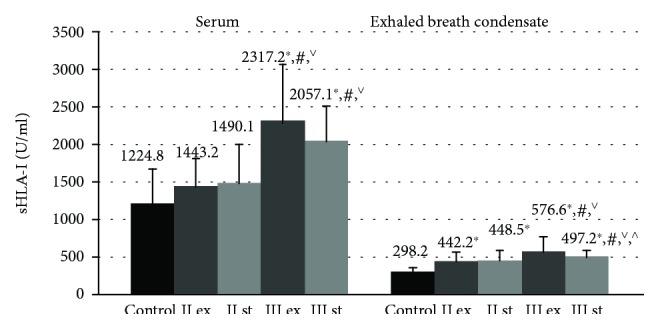
The concentration of sHLA-I molecules in blood serum and exhaled breath condensate in COPD patients during the exacerbation and in the stable period. Data are presented as mean ± SD. Control: healthy nonsmoking volunteers, II: moderate COPD, III: severe COPD, ex: exacerbation, and st: stable phase. ^∗^*p* < 0.05 versus healthy nonsmokers, ^#^*p* < 0.05 versus patients with moderate COPD during the exacerbation, ^∨^*p* < 0.05 versus patients with moderate COPD in the stable phase, and ^^^*p* < 0.05 versus patients with severe COPD during the exacerbation.

The content of sHLA-I in EBC in all examined patients with COPD was significantly higher than in healthy nonsmoking volunteers (298.2 ± 62.32 U/ml) (Figure 1).

The endobronchial concentrations of sHLA-I in patients with moderate COPD both in the stable phase (448.5 ± 84.5 U/ml) and during the exacerbation (442.2 ± 94.3 U/ml) were lower than in patients with the severe disease (*р* < 0.05).

In the GOLD III in exacerbation, the level of sHLA-I proteins in EBC (576.6 ± 158.1 U/ml) was statistically increased compared to severe clinically stable COPD (497.2 ± 86.2 U/ml, *p* = 0.032).

The serum concentrations of sHLA-I in patients with moderate COPD in the stable phase (1443.2 ± 369.9 U/ml) and during exacerbation (1490.1 ± 508.5 U/ml) did not differ from the control values (1224.8 ± 451.2 U/ml, *p* > 0.05) (Figure 1).

The level of sHLA-I in blood serum in all patients with severe COPD was statistically significantly higher than that of nonsmoking volunteers and GOLD II patients (*p* ≤ 0.001).

**Figure 2:**
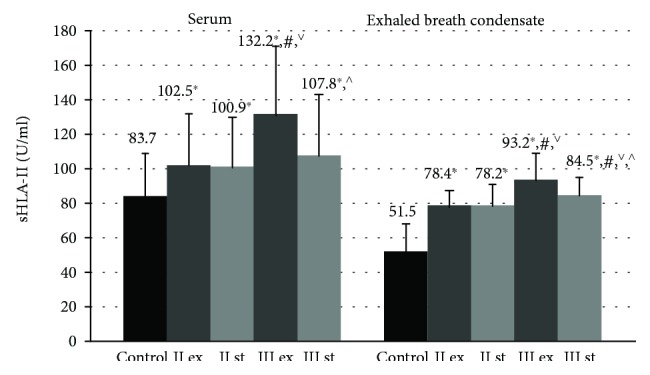
The concentration of sHLA-II molecules in blood serum and exhaled breath condensate in COPD patients during the exacerbation and stable period. Data are presented as mean ± SD. Control: healthy nonsmoking volunteers, II: moderate COPD, III: severe COPD, ex: exacerbation, and st: stable phase. ^∗^*p* < 0.05 versus healthy nonsmokers, ^#^*p* < 0.05 versus patients with moderate COPD during the exacerbation, ^∨^*p* < 0.05 versus patients with moderate COPD in the stable phase, and ^^^*p* < 0.05 versus patients with severe COPD during the exacerbation.

The serum and endobronchial concentrations of sHLA-II were statistically significantly higher in all the examined patients compared to the control group (1224.8 ± 451.2 and 298.2 ± 62.32, respectively, *p* ≤ 0.001) (Figure 2).

The content of sHLA-II molecules in EBC in GOLD II was lower than in severe disease (*p* < 0.05). At the same time, in GOLD III patients during the exacerbation, the concentration of sHLA-II molecules in EBC (93.2 ± 15.6 U/ml) was statistically significantly increased compared to the stable period of the disease (84.5 ± 10.04 U/ml, *p* = 0.032).

The serum level of sHLA-II proteins in healthy nonsmoking volunteers was 83.7 ± 25.3 U/ml. The highest concentration of sHLA-II in serum was detected in patients with severe COPD in the exacerbation period compared to other individuals examined (*p* < 0.05). Besides, in GOLD III patients during the exacerbation, the serum level of sHLA-II (132.2 ± 39.2 U/ml) exceeded the values of the stable phase of the disease (107.8 ± 35.4 U/ml, *p* = 0.034).

The association analysis revealed no statistically significant correlations between sHLA-I and sHLA-II levels in the tested biological fluids with moderate COPD (Table 2).

At the same time, in severe COPD, the positive associations between serum levels of sHLA-I and sHLA-II have been found (Table 3).

In addition, the positive correlations between serum and endobronchial concentrations for both sHLA-I and sHLA-II were also determined in GOLD III patients.

The association analysis between the spirometric parameters and the content of the studied soluble molecules revealed that in patients with moderate COPD there were no significant correlations between serum and endobronchial content of sHLA-I molecules and measures of lung function (Table 4).

In severe COPD, an inverse relationship between the lung function parameters such as FEV_1_, IC, and the serum level of sHLA-I (*r* = 0.51, *p* = 0.01 and *r* = −0.63, *p* = 0.01, respectively) was determined.

A decrease in investigated spirometric parameters in patients was accompanied by an increase in the endobronchial content of sHLA-I and sHLA-II proteins in GOLD III patients.

The correlation analysis showed a negative relationship between the serum content of sHLA-II and FEV_1_ and FEV_1_/FVC in moderate COPD and a negative associations between the serum content of sHLA-II and all investigated lung function parameters in GOLD III.

## 4. Discussion

In this study, we established an increase in the level of sHLA-I and sHLA-II molecules in EBC as well as an elevated serum level of sHLA-II in all studied patients compared to healthy nonsmoking volunteers.

Significant differences in the concentration of sHLA-I and sHLA-II molecules of the studied biological fluids in GOLD II patients in the stable phase and during exacerbation were not revealed. Thus, the content of these molecules in moderate COPD did not depend on the period of the disease.

The maximum accumulation of sHLA-I and sHLA-II molecules in both the respiratory tract and circulation was recorded in severe COPD during the exacerbation. Besides, in patients with exacerbation of GOLD III, the serum content of sHLA-II and the endobronchial levels of sHLA-I and sHLA-II were significantly higher than in severe clinically stable COPD. The revealed differences in the content of soluble molecules of the major histocompatibility complex in COPD patients depending on the severity degree and the disease period allow us to consider these proteins as potential biomarkers of activation of local and systemic inflammation and severity of COPD.

The established increase in the concentration of soluble sHLA-I and sHLA-II in the examined patients may be caused by both intensive proteolytic shedding and increased secretion of these molecules due to alternative splicing by activated cells producing these proteins. It is known that sHLA-I molecules are produced by T and B lymphocytes [23]. Dendritic cells and macrophages, lymphocytes, and endothelial and epithelial cells can be sources of sHLA-II proteins [12]. These cells under the conditions of chronic inflammation in COPD patients are in a permanently activated state. In this case, the increased content of sHLA-I and sHLA-II molecules in the tested biological fluids can reflect the activity of both local and systemic inflammatory process in patients with COPD.

The positive correlations between the content of sHLA-I and sHLA-II in serum and EBC in GOLD III indicate the identity of the mechanisms that potentiate the production of these soluble molecules in expressed local and systemic inflammation and hypoxemia.

Soluble molecules of the major histocompatibility complex are intercellular protein communicators and occupy one of the important positions in the realization of the immune response [16, 17, 35–38]. sHLA-I and sHLA-II molecules binding to their own physiological ligands (CD8 and CD4 molecules) are capable of suppressing the functional activity of T lymphocytes and natural killers through receptor blockade and/or inducing the secretion of a soluble Fas ligand which stimulates apoptosis of these cells. It is known, that the main function of CD4+ and CD8+ T cells, as well as NK cells, is antiviral and antibacterial protection. In this case, the increased formation of sHLA-I and sHLA-II molecules in COPD patients may be a cause of disruption in the presentation processes of antigens and an inadequate immune response concerning infectious agents which play a key role in the development of disease exacerbation.

Furthermore, it is known that soluble molecules of the major histocompatibility complex can be phagocytosed by antigen-presenting cells, degraded to peptides, and presented to CD4+ T cells in the context of membrane antigens of HLA class II. The latter process is known as an indirect presentation and can lead to immune tolerance and, ultimately, to a distortion of the protective function of the immune system [39]. Thus, the increase in the content of sHLA-I and sHLA-II molecules may be an unfavorable prognostic factor and one of the reasons for the intensification and progression of chronic inflammation in COPD patients.

The established correlations between the serum level of sHLA-II and lung function parameters in all examined patients with COPD indicate the potential participation of these molecules in the progression of systemic inflammation, as well as their influence on the pathophysiological mechanisms of bronchial patency disorders. The obtained results allow us to consider serum levels of sHLA-II as possible systemic markers of COPD severity.

In our work, we found that in patients with severe COPD, an increase in the content of sHLA-I in circulation and EBC, the raise of endobronchial concentration of sHLA-II occurred against the background of a decrease in FEV_1_ and FEV_1_/FVC. The findings suggest a possible role of these proteins in the pathogenesis of airway obstruction progression, as well as in the formation of fibrosis processes and lung tissue remodeling in expressed inflammation in COPD patients.

Moreover, in severe COPD, the negative associations between the serum and endobronchial content of the studied soluble molecules and inspiratory capacity (IC) were detected. The decrease in the IC parameter characterizes the development of lung hyperinflation (LH). This pathophysiological disorder in patients with COPD is associated with the loss of elasticity of the lung tissue, bronchoconstriction, and subsequent development of hypoxia. Thus, the revealed correlations may indicate the relationship between high concentrations of circulating and endobronchial sHLA-I and sHLA-II molecules with structural and functional changes in the respiratory tract, as well as with the development of hypoxemia and the disturbance of gas metabolism under conditions of severe inflammation in COPD.

## 5. Conclusion

Summarizing the obtained results, we can conclude that sHLA-I and sHLA-II molecules make a significant contribution to the development and progression of local and systemic inflammation in COPD.

Exacerbation in patients with severe COPD was characterized by a maximum increase in the level of sHLA-I and sHLA-II in serum and respiratory tract. These results allow us to consider the high content of these molecules as an unfavorable prognostic factor and a potential monitoring marker of the intensification of chronic inflammation in COPD.

The revealed associations between the lung function parameters and the serum concentration of sHLA-II in all examined patients with COPD make it possible to use the level of these proteins as additional systemic indicators of the disease severity.

## Figures and Tables

**Table 1:** Characteristics of patients with COPD and healthy nonsmokers included in the study.

		COPD	
	Healthy nonsmokers	Moderate	Severe
Subjects (*n*)	21	52	53
Age (years)	51.3 ± 8.5	53.1 ± 5.7	57.1 ± 5.1
Smoking pack-years	0	35.4 ± 6.7	41.3 ± 5.8
FEV_1_% pred	103.2 ± 4.1	61.4 ± 5.1	39.4 ± 4.1
FEV_1_/FVC %	105.3 ± 3.5	62.1 ± 3.8	48.7 ± 8.1
Inspiratory capacity IC (%)	109.2 ± 2.3	67.6 ± 6.9	64.1 ± 7.1

Data were presented as mean ± SD. COPD: chronic obstructive pulmonary disease; pack-years: number of cigarette packs per day multiplied by the number of smoking years; FEV_1_: forced expiratory volume in one second; % pred: % predicted; FVC: forced vital capacity; IC: inspiratory capacity (%).

**Table 2:** Correlations between sHLA-I and sHLA-II levels in biological fluids in patients with moderate COPD.

	sHLA-II EBC	sHLA-I serum	sHLA-I EBC
sHLA-I EBC	*r* = 0.13*p* = 0.63	*r* = 0.22*p* = 0.44	—
sHLA-I serum	*r* = 0.1*p* = 0.77		—
sHLA-II serum	*r* = 0.26*p* = 0.23	*r* = 0.25*p* = 0.24	*r* = 0.17*p* = 0.52

*r*: correlation coefficient.

**Table 3:** Correlations between sHLA-I and sHLA-II levels in biological fluids in patients with severe COPD.

	sHLA-II EBC	sHLA-I serum	sHLA-I EBC
sHLA-I EBC	*r* = 0.06*p* = 0.77	**r** = 0.51**p** = 0.01	—
sHLA-I serum	*r* = 0.1*p* = 0.66	—	—
sHLA-II serum	**r** = 0.45**p** = 0.02	**r** = 0.42**p** = 0.029	*r* = −0.14*p* = 0.47

*r*: correlation coefficient.

**Table 4:** Correlations between the lung function parameters and the concentrations of sHLA-I and sHLA-II molecules in COPD patients.

	GOLD II			GOLD III		
	FEV_**1**_**(%)**	FEV_1_/FVC (%)	IC (%)	FEV_1_ (%)	FEV_1_/FVC (%)	IC (%)
sHLA-I (EBC)	*r* = −0.33*p* = 0.19	*r* = −0.25*p* = 0.34	*r* = 0.23*p* = 0.41	**r** = −0.48**p** = 0.03	**r** = −0.49**p** = 0.02	**r** = −0.71**p** = 0.003
sHLA-I (serum)	*r* = −0.17*p* = 0.5	*r* = −0.19*p* = 0.38	*r* = −0.27*p* = 0.3	**r** = −0.51**p** = 0.01	*r* = −0.33*p* = 0.22	**r** = −0.63**p** = 0.01
sHLA-II (EBC)	*r* = 0.23*p* = 0.41	*r* = 0.12*p* = 0.64	*r* = 0.3*p* = 0.12	**r** = −0.39**p** = 0.04	**r** = −0.43**p** = 0.03	**r** = −0.59**p** = 0.016
sHLA-II (serum)	**r** = −0.55**p** = 0.01	**r** = −0.45**p** = 0.03	*r* = −0.11*p* = 0.67	**r** = −0.61**p** = 0.001	**r** = −0.47**p** = 0.02	**r** = −0.76**p** = 0.001

*r*: correlation coefficient; EBC: exhaled breath condensate; FEV_1_: forced expiratory volume in 1 second; % pred: % predicted; FVC: forced vital capacity; IC: inspiratory capacity (%).

## Data Availability

The data used to support the findings of this study are available from the corresponding author upon request.
